# Molecular Evaluation of Rifampicin and Isoniazid Resistance in Pulmonary Tuberculosis Using Cartridge-Based Nucleic Acid Amplification Test and Line Probe Assay

**DOI:** 10.7759/cureus.110237

**Published:** 2026-06-04

**Authors:** Rinku Yadav, Rosy Bala, Aparna Parmar, Parminder Singh, Aman Singh, Monika Sharma, Anshu Singh, Pawan Kalkal

**Affiliations:** 1 Microbiology, Maharishi Markandeshwar Institute of Medical Sciences and Research, Maharishi Markandeshwar (Deemed to be University) Mullana, Ambala, IND; 2 Microbiology, Pandit Bhagwat Dayal Sharma Post Graduate Institute of Medical Sciences, Rohtak, IND; 3 Orthopaedics, Adesh Medical College & Hospital, Mohri, IND; 4 Pharmacology, Pandit Neki Ram Sharma Government Medical College, Bhiwani, IND; 5 Microbiology, All India Institute of Medical Sciences, Rishikesh, Rishikesh, IND; 6 Oncology, Positron Super Speciality and Cancer Hospital, Rohtak, IND

**Keywords:** drug-resistant tuberculosis, isoniazid resistance, katg mutation, line probe assay (lpa), multidrug-resistant tuberculosis (mdr-tb), rifampicin resistance, rpob mutation

## Abstract

Background

Pulmonary tuberculosis (TB) continues to be a major public health problem, especially with the rising number of drug-resistant TB cases. Rapid molecular tests such as cartridge-based nucleic acid amplification test (CB-NAAT) and line probe assay (LPA) help in the early diagnosis of TB and detection of drug resistance, allowing timely treatment and better disease control.

Methods

This cross-sectional study included 3451 pulmonary samples collected from patients suspected of having pulmonary tuberculosis. All samples were tested by CB-NAAT for detection of *Mycobacterium tuberculosis* and rifampicin resistance. Samples showing drug resistance were further analyzed by first-line LPA to identify genetic mutations associated with rifampicin and isoniazid resistance.

Results

Among the 3451 pulmonary samples tested, *M. tuberculosis* was detected in 758 (21.9%) cases, while in 2682 (77.7%) samples, it was not detected. Invalid/error results were observed in only 11 (0.3%) samples. Rifampicin resistance was identified in 42 (5.5%) isolates. Sputum was the most common positive sample, accounting for 661 (87.2%) cases. Most patients were newly diagnosed TB cases (n=662, 87.3%), whereas previously treated cases, TB contacts, and HIV-positive patients constituted a smaller proportion. First-line LPA detected multidrug-resistant tuberculosis (MDR-TB) in 29 isolates. The most common mutation associated with rifampicin resistance was *rpoB* MUT3 (S531L/H531L), while *katG* MUT1 (S315T1) was the predominant mutation linked to isoniazid resistance.

Conclusion

CB-NAAT and LPA proved to be useful and reliable methods for rapid diagnosis of pulmonary tuberculosis and detection of drug resistance. Early identification of drug-resistant TB through molecular techniques can help in timely treatment initiation and improved tuberculosis control. The predominance of rpoB S531L/H531L and katG S315T1 mutations suggests their important role in the development of anti-tubercular drug resistance.

## Introduction

The *Mycobacterium tuberculosis* bacterium (MTB) is the cause of tuberculosis (TB), one of the oldest diseases affecting humans. In the 21st century, TB is still a major global public health concern. The emergence of increasingly drug-resistant strains of the disease poses a significant challenge to current and future efforts to control tuberculosis [1,2].

According to the India TB Report 2024, a record high of 25.2 lakh TB cases, including 63,929 multidrug-resistant/rifampicin-resistant (MDR/RR)-TB cases, were reported in India in 2023 [3]. The country has the highest annual number of newly diagnosed cases and accounts for almost 25% of the global TB burden.

Drug-resistant tuberculosis is the biggest threat to the advancements in tuberculosis control. MDR TB is characterised by resistance to both isoniazid and rifampicin. For MDR TB, a different treatment strategy utilising second-line anti-TB drugs is necessary. Because conventional culture and drug susceptibility testing (DST) require an extended diagnosis period of six to eight weeks, patients who receive an inappropriate medication regimen continue to spread drug-resistant mutant strains throughout the community [4].

The world's top priorities for TB care and control are increasing case detection and guaranteeing early diagnosis of cases. The Xpert MTB/RIF assay (Cepheid, Sunnyvale, California, United States) plays a crucial role in the timely diagnosis of TB, enabling early and appropriate treatment by simultaneously identifying *M. tuberculosis* and rifampicin resistance within two hours with high sensitivity and specificity [5].

Multidrug resistance is caused by random mutations, mainly in the genes *rpoB*, *KatG*, and *inhA*. This is exploited by genotypic or molecular approaches. Early resistance diagnosis and appropriate treatment can be initiated by identifying these mutations. The line probe assay (LPA) is a promising rapid diagnostic method that combines the multiplex polymerase chain reaction and the DNA strip reverse hybridisation assay [6]. The World Health Organization (WHO) suggests rapid molecular tests like cartridge-based nucleic acid amplification test (CB-NAAT) (GeneXpert MTB/RIF) and LPA as frontline screening tools to identify MDR-TB. When compared to conventional culture methods, these tests significantly shorten diagnosis times. Although little is known about the efficacy of LPAs in India, these tests can identify isoniazid and rifampicin resistance in a matter of days, which is crucial for halting transmission and initiating prompt, effective treatment, especially in high-burden countries [7].

The aim of this study was to determine the genotypic characterization and drug-resistance profile of *M. tuberculosis* isolates from clinically diagnosed pulmonary TB patients. The study also aimed to detect resistance to first-line anti-tubercular drugs and determine the prevalence of MDR-TB among suspected pulmonary TB cases.

## Materials and methods

This was a cross-sectional collaborative study conducted between September 2024 and February 2026 in the Culture and Drug Susceptibility Testing (C&DST) Laboratory, Pandit Bhagwat Dayal Sharma Post Graduate Institute of Medical Sciences, Rohtak, Haryana, India, and the Department of Microbiology, Maharishi Markandeshwar Institute of Medical Sciences and Research, Mullana, Ambala, Haryana, India. The study was approved by the Institutional Ethical Committee of the Maharishi Markandeshwar Institute of Medical Sciences and Research (project number: IEC-3143).

Sample collection and inclusion

Pulmonary samples were collected from patients clinically suspected of TB. Two sputum specimens were collected from each patient in sterile Falcon tubes and transported promptly to the laboratory for processing. In cases of delay, the specimens were stored at 4°C until further processing.

Only samples with a volume of ≥2 mL collected in sterile, wide-mouth, leak-proof Falcon tubes were considered eligible for analysis. Extra-pulmonary specimens, samples with a volume of <2 mL, and non-tuberculous mycobacterial (NTM) isolates were excluded from the study. Samples with indeterminate or invalid results were excluded from the final analysis.

Laboratory processing

Pulmonary specimens were analysed using CB-NAAT (GeneXpert MTB/RIF assay) for the detection of *M. tuberculosis* and rifampicin resistance. All pulmonary tuberculosis samples deemed positive by CB-NAAT were further subjected to first-line LPA (GenoType MTBDRplus VER 2.0; Hain Lifescience GmbH, Nehren, Germany), which was performed according to standard laboratory procedures.

Sputum samples were first subjected to digestion and decontamination using the N-acetyl-L-cysteine (NALC)-sodium hydroxide (NaOH) method. An equal volume of NaOH-NALC-sodium citrate solution was added to the sputum sample in a 50 mL centrifuge tube, followed by gentle vortexing or hand mixing for 15-30 seconds. The mixture was allowed to stand for 15-20 minutes with intermittent mixing every 5-10 minutes to ensure complete liquefaction. Phosphate buffer (pH 6.8) was then added up to the 50 mL mark, mixed thoroughly, and centrifuged at 3000 g for 15-20 minutes at 4 °C. After allowing aerosols to settle for five minutes, the supernatant was discarded, and the sediment was resuspended in 1-2 mL phosphate buffer (pH 6.8). The pellet was further homogenised in 1-1.5 mL of phosphate buffer, and aliquots were prepared. The processed sediment was used for inoculation into Mycobacteria Growth Indicator Tubes (MGIT) and for DNA extraction for LPA. Ziehl-Neelsen (ZN) staining was performed after decontamination. All smear-positive samples were processed for DNA extraction, while smear-negative samples were inoculated into MGIT tubes for mycobacterial growth detection.

DNA Extraction

DNA extraction was carried out using the GenoLyse® kit (Bruker Corporation, Billerica, Massachusetts, United States), which lyses the mycobacterial cell wall to release genomic DNA. Approximately 500 µL of the decontaminated specimen was transferred into a screw-cap microcentrifuge tube and centrifuged to pellet the bacteria. The pellet was resuspended in 100 µL of lysis buffer (A-LYS) and vortexed thoroughly. The suspension was incubated at 95°C for 15-20 minutes for heat inactivation and cell lysis. Subsequently, 100 µL of neutralisation buffer (A-NB) was added to stabilise the pH. The mixture was vortexed briefly and centrifuged at high speed (9600-13,000 g) for 5-10 minutes. The supernatant containing extracted mycobacterial DNA was carefully transferred to a sterile microcentrifuge tube. Extracted DNA was stored at 2-8°C for short-term use and at −20°C for long-term preservation.

Amplification of Extracted DNA

DNA amplification was performed according to the manufacturer’s protocol. All reagents were stored at 2-8°C, while amplification mixes A and B were maintained at −20°C until use. A master mix volume of 45 µL was prepared for each specimen, consisting of 10 µL Amplification Mix A (AM-A) and 35 µL Amplification Mix B (AM-B). The master mix was prepared according to the number of samples processed in each run. For DNA amplification, 5 µL of extracted DNA was added to 45 µL of prepared master mix, making the final reaction volume 50 µL per specimen. The amplification of DNA was then carried out using a polymerase chain reaction (PCR) machine.

Hybridization and Interpretation of Resistance-Associated Mutations

Following PCR amplification, the amplified products were subjected to reverse hybridization using GenoType MTBDRplus VER 2.0 strips according to the manufacturer's instructions. The strips were subsequently washed, incubated with conjugate, and developed for visualization of hybridization bands. The resulting banding patterns were interpreted using the manufacturer's evaluation chart to identify *M. tuberculosis* complex and mutations associated with rifampicin and isoniazid resistance.

Positive and negative controls were included in each PCR, and LPA was run in accordance with the manufacturer's guidelines to ensure the validity and quality of the assay results.

Data analysis

All data were compiled and analysed using Microsoft Excel (Microsoft Corporation, Redmond, Washington, United States). Descriptive statistical analysis was performed for the study. The results were expressed in terms of frequencies, percentages, and proportions.

## Results

A total of 3,451 suspected pulmonary TB cases were screened, of which 758 (21.9%) were positive by CB-NAAT, while invalid/error results were observed in only 11 (0.3%) samples. Among the 758 CB-NAAT-positive samples, sputum was the most common specimen type, accounting for 661 (87.2%) cases, followed by bronchoalveolar lavage (BAL) in 74 (9.7%) cases, endotracheal aspirates in 17 (2.2%) cases, and gastric aspirates in nine (1.1%) cases. Newly diagnosed TB cases constituted the majority (n=662, 87.3%), while previously treated/treatment-failure cases accounted for 48 (6.3%); TB contact history and HIV seropositivity were observed in 35 (4.6%) and 13 (1.7%) cases, respectively. Rifampicin resistance profiling by CB-NAAT showed that 709 (93.5%) TB isolates were rifampicin sensitive, while 42 (5.5%) were rifampicin resistant; indeterminate results were observed in seven (0.9%) samples (Table 1).

**Table 1:** Distribution of sample types, clinical history, and rifampicin resistance among CB-NAAT positive tuberculosis cases (N = 758) CB-NAAT: cartridge-based nucleic acid amplification test

Category	Variable	Frequency	Percentage
Sample Type	Sputum	661	87.2
	Bronchoalveolar Lavage (BAL)	74	9.7
	Endotracheal Aspirate	17	2.2
	Gastric Aspirate	9	1.1
Clinical History	New TB Cases	662	87.3
	Previously Treated TB	48	6.3
	Contact with TB Patients	35	4.6
	HIV Positive Status	13	1.7
Rifampicin Resistance Pattern	Rifampicin Sensitive	709	93.5
	Rifampicin Resistant	42	5.5
	Rifampicin Indeterminate	7	0.9
	Total	758	100

First-line LPA demonstrated that most TB isolates were sensitive to both rifampicin and isoniazid (n=674), while rifampicin monoresistance and MDR-TB were observed in 14 and 29 isolates, respectively. Among isoniazid-resistant isolates, *KatG* mutations predominated over *inhA* mutations. Mutation analysis of MDR-TB isolates revealed that *rpoB* MUT3 (S531L) was the predominant rifampicin resistance-conferring mutation, detected in 9/14 (64.3%) rifampicin-monoresistant isolates and 23/29 (79.3%) MDR-TB isolates. Less frequent mutations included *rpoB* MUT2B (S526D) and MUT1 (D516V), while a few isolates showed loss of wild-type bands without corresponding mutant bands, indicating uncommon or unknown mutations. Among isoniazid-resistant TB isolates, *KatG* MUT1 (S315T1) was the predominant mutation, detected in 25/29 (86.2%) MDR-TB isolates and 29 isoniazid-monoresistant isolates, whereas *KatG *MUT2 (S315T2) was observed less frequently. In the *inhA* gene region, the MUT1 (C15T) mutation was identified in 2/29 (6.9%) MDR-TB isolates and 11 isoniazid-monoresistant isolates, as shown in Table 2.

**Table 2:** Distribution of first-line LPA mutation patterns in Mycobacterium tuberculosis LPA: line probe assay; Rif: rifampicin; WT: wild type; MUT: mutation

Gene	Band type	Mutation Pattern	Rif monoresistance, n (%)	MDR, n (%)	Mono isonized resistance, n (%)
rpoB	WT missing + MUT present	rpoB MUT3 (S531L)	9 (64.3%)	23 (79.3%)	-
	WT missing + MUT present	rpoB MUT2B (S526D)	2 (14.3%)	1 (3.4%)	-
	WT missing + MUT present	rpoB MUT 1 (D516V)	1 (7.1%)	2 (6.9%)	-
	All WT present + MUT present	rpoB MUT3 (S531L)	1 (7.1%)	-	-
	WT missing only	Unknown mutation	1 (7.1%)	3 (10.3%)	-
KatG	WT missing + MUT present	MUT -1 S315T1	-	25 (86.2%)	29 (69%)
		MUT -2 S315T2	-	2 (6.9%)	2 (4.8%)
inhA	WT missing + MUT present	MUT -1 C15T	-	2 (6.9%)	11 (26.2%)
Total			14 (100%)	29 (100%)	42 (100%)

## Discussion

The present study reported MTB positivity in 21.9% of pulmonary specimens by CB-NAAT, which was lower than the positivity rates reported by Bisht et al. [2] and Bhatnagar et al. [8], but higher than that observed by Hakkak et al. [9]. Variations in MTB positivity among different studies may be attributed to differences in sample size, geographical distribution, patient demographics, burden of TB, and healthcare-seeking behaviour. Despite these differences, the findings of the present study reaffirm the importance of CB-NAAT as a rapid, sensitive, and reliable molecular diagnostic tool for the early detection of pulmonary TB. The assay not only reduces the turnaround time for diagnosis but also facilitates prompt initiation of treatment, thereby limiting disease transmission and improving patient outcomes.

In the present study, sputum was the most common CB-NAAT-positive specimen, accounting for 87.2% of all positive samples, followed by BAL (9.7%) and gastric aspirates (1.1%). The predominance of sputum positivity may be due to its routine use as the primary specimen for pulmonary TB diagnosis and its higher bacillary load compared to other respiratory samples. However, studies conducted by Mukharya et al. [10], Kumar et al. [11], and Maheshwari et al. [12] reported comparatively higher positivity rates in BAL and gastric aspirate specimens. These differences may reflect variations in patient age groups, clinical presentation, specimen collection practices, and inclusion of patients unable to produce sputum. Nevertheless, the present findings highlight sputum as the most practical and effective specimen for CB-NAAT-based pulmonary TB diagnosis in routine clinical settings.

Analysis of demographic and clinical risk factors revealed that newly diagnosed TB cases constituted the majority of patients (87.3%), whereas previously treated cases accounted for 6.3%, history of TB contact was observed in 4.6%, and HIV seropositivity was noted in 1.7% of patients. The predominance of newly diagnosed cases suggests ongoing transmission of TB within the community and emphasises the need for early screening and detection strategies. Similar observations were reported by Singh et al. [1], who also documented higher rates of drug resistance among previously treated patients, individuals with a history of TB contact, and HIV-positive patients. Previously treated patients are known to have an increased risk of developing drug resistance due to incomplete therapy, treatment failure, relapse, or poor treatment adherence. Likewise, HIV infection contributes to immunosuppression, increasing susceptibility to TB and complicating disease management. These findings underline the importance of targeted surveillance and resistance screening in high-risk groups.

Rifampicin resistance profiling by CB-NAAT demonstrated that 709 (93.5%) isolates were rifampicin-sensitive, 42 (5.5%) were rifampicin-resistant, and seven (0.9%) showed indeterminate results. These findings are comparable to those reported by Prathiksha et al. [13] and Devrari and Chauhan [14]. Rifampicin resistance is considered a surrogate marker for MDR-TB, making its rapid detection clinically significant. The high proportion of rifampicin-sensitive isolates indicates that drug-sensitive TB remains predominant in the study population. However, the detection of rifampicin-resistant cases highlights the continuing challenge posed by MDR-TB. The small proportion of indeterminate results may be attributed to low bacillary load, technical limitations, or poor sample quality. Overall, these findings emphasise the effectiveness of CB-NAAT as an essential diagnostic modality for rapid detection of rifampicin resistance and early identification of MDR-TB, thereby enabling timely initiation of appropriate anti-tubercular therapy.

Mutation analysis using the LPA revealed that the most common mutation associated with rifampicin resistance was *rpoB* MUT3 (S531L), detected in 64.3% of rifampicin monoresistant isolates and 79.3% of MDR-TB isolates. Other mutations identified included *rpoB* MUT2B (S526D) and *rpoB* MUT1 (D516V), although these occurred less frequently. Additionally, a few isolates demonstrated loss of wild-type bands without corresponding mutant bands, indicating the presence of uncommon or unknown mutations outside the commonly targeted regions. The predominance of the S531L mutation observed in the present study is consistent with findings reported by Maurya et al. [15] and Madhuri et al. [4]. The S531L mutation is one of the most frequently encountered mutations within the rifampicin resistance-determining region (RRDR) of the* rpoB* gene and is strongly associated with high-level rifampicin resistance.

Among isoniazid-resistant isolates, the most frequent mutation was *KatG* MUT1 (S315T1), observed in 69.0% of isoniazid monoresistant isolates and 86.2% of MDR-TB isolates. The *KatG* MUT2 (S315T2) mutation was detected less commonly, while the *inhA* MUT1 (C15T) mutation was identified in 26.2% of isoniazid monoresistant isolates and 6.9% of MDR isolates. Mutations in the *KatG* gene are generally associated with high-level isoniazid resistance, whereas mutations in the *inhA* promoter region are linked to low-level resistance and cross-resistance to ethionamide. The predominance of the *KatG* S315T1 mutation in the present study correlates with observations made by previous researchers, including Maurya et al. [15] and Madhuri et al. [4]. These findings suggest that KatG-mediated resistance remains the principal mechanism of isoniazid resistance in the study population.

Overall, the present study highlights the significant role of CB-NAAT and LPA in the rapid diagnosis of TB and the detection of drug resistance-associated mutations. Early identification of rifampicin and isoniazid resistance is crucial for timely initiation of effective treatment regimens, prevention of transmission, and improved management of drug-resistant TB. The predominance of *rpoB* S531L and *KatG* S315T1 mutations observed in the present study also provides valuable epidemiological insight into the molecular basis of drug resistance in the regional population.

Limitations of the study

The study included only pulmonary TB specimens, while extrapulmonary TB cases were excluded. Therefore, the mutation patterns and drug resistance profiles observed in this study may not fully represent extrapulmonary TB cases.

## Conclusions

The present study demonstrated that CB-NAAT is a rapid and reliable diagnostic tool for the detection of *M. tuberculosis* and rifampicin resistance in pulmonary tuberculosis patients. Sputum was identified as the most effective specimen for MTB detection. The majority of cases were newly diagnosed tuberculosis patients, while rifampicin resistance was observed in a smaller proportion of cases. The LPA further identified *rpoB *MUT3 (S531L/H531L) and *KatG* MUT1 (S315T1) as the most common mutations associated with rifampicin and isoniazid resistance, respectively. Early molecular diagnosis using CB-NAAT and LPA can aid in prompt detection and management of MDR-TB.
